# Sociodemographic and medical characteristics of liver cirrhosis deaths in a Ghanaian tertiary hospital

**DOI:** 10.4314/gmj.v56i4.4

**Published:** 2022-12

**Authors:** William K Agbozo, Bartholomew Dzudzor, Eric NY Nyarko, Karen Lartey-Abrahams, Roberta N A Mensah, Kenneth Tachi

**Affiliations:** 1 Department of Physician Assistantship, School of Medicine, and Health Sciences, Central University, Ghana; 2 Department of Medical Biochemistry, University of Ghana Medical School, Ghana; 3 Department of Chemical Pathology, University of Ghana Medical School, Ghana; 4 West African Centre for Cell Biology of Infectious Pathogens, University of Ghana, Ghana; 5 Gastroenterology Unit, Korle-Bu Teaching Hospital, Ghana; 6 Department of Medicine & Therapeutics, University of Ghana Medical School, Ghana

**Keywords:** Liver diseases, Cirrhosis, Chronic alcoholism, HBV, Ghana

## Abstract

**Objective:**

Cirrhosis is common in Ghana because of its high risk factors prevalence. However, information on cirrhosis in Ghana is lacking. This study aimed to study the clinical, and laboratory characteristics of cirrhotic patients in a tertiary hospital in Ghana.

**Design:**

This was a retrospective study of sociodemographic characteristics, symptoms and signs, biochemical and fibrotic indices, treatments, and complications data of 247 patients with cirrhosis who died on admission.

**Setting:**

This study was carried out at the Gastroenterology Unit of the Korle-Bu Teaching Hospital, Ghana,

**Results:**

Two-thirds (68.0%) of the patients were within 30 to 60 years, with more than half (73.7%) being males. The most common aetiological factors among the patients were Hepatitis B virus infection (53.8%), alcohol use (31.6%) and Hepatitis C virus infection (4.9%). More than half (55.0%) of the patients reported late for admission, and 67.2% died within the first two weeks of admission. The most common clinical feature was abdominal distension (61.1% of patients), and the least was upper-abdominal mass (14.2%). The levels of most liver test parameters were elevated, fibrotic indices were high, and haemoglobin and albumin levels were reduced. More than half (53.8%) of the patients were in Child Pugh class B. The most common complication was hepatic encephalopathy; the least was hepato-renal syndrome. Definite treatment for complications of cirrhosis was lacking.

**Conclusion:**

Deaths from cirrhosis at the hospital were mostly of young males with chronic hepatitis B infection. Implementation of hepatitis B prevention and treatment guidelines can help reduce cirrhosis deaths.

**Funding:**

None declared

## Introduction

Liver cirrhosis is one of the major complications of chronic liver disease. It is characterised by recurrent and repeated inflammation and healing of the liver, which leads to fibrosis, scarring and nodule (both macro and micro) formation culminating into malfunctioning hepatic histoarchitecture.^1^ This can lead to liver failure and patient death, especially if a liver transplant is not received. Worldwide, it is estimated that 25 of every 1000 deaths are attributable to cirrhosis.^2^ In 2017 alone, cirrhosis accounted for 1,320,000 deaths globally with two-thirds being males.^2^

Several causes of liver cirrhosis are reported in the literature.^3^–^7^ These include excessive alcohol use and chronic viral hepatitis B and C infections, non-alcoholic fatty liver disease (NAFLD) and to a lesser extent, haemochromatosis, Wilson's disease, α1- antitrypsin deficiency and biliary disorders.^3^–^8^ The prevalence of these causes shows socio-geographic variations. Chronic excess alcohol use is the leading cause worldwide, accounting for over half of the world's cirrhosis.^3^,^7^ Excess alcohol use is also the major cause of cirrhosis in the West, including most European countries.^3^,^7^ However, in sub-Saharan Africa and Asia, the major cause of cirrhosis is chronic viral hepatitis, especially hepatitis B virus (HBV).^2^

The socioeconomic burden and the high mortality rate of cirrhosis cannot be overemphasised. The World Health Organization (WHO) has identified sub-Saharan Africa as the region with the highest prevalence, morbidity and mortality from cirrhosis and Egypt as the country with the highest mortality rate.^2^ Between 1980 and 2010, cirrhosis-related deaths doubled in sub-Saharan Africa.^9^ Despite this increasing burden, cirrhosis remains a neglected disease in the sub-region.^10^ Thirty percent of causes remain unknown, with a likelihood of underestimation of the role of non-alcoholic fatty liver disease (NAFLD).^10^ Treatment for viral hepatitis B and C infections are expensive, and that of liver cirrhosis is inaccessible in most parts of the sub-region because of the lack of facilities for liver transplantation and the huge shortage of hepatologists and gastroenterologists, interventional radiologists, hepatobiliary surgeons, and pathologists. Again, more than 50% of patients are admitted to hospitals with end-stage disease, and mortality is high at initial hospitalization.^11^

Liver cirrhosis is common in Ghana because of the high prevalence of risk factors; chronic hepatitis B virus (HBV), hepatitis C virus (HCV), and alcohol use.^4^–^6^ Despite the high prevalence of these risk factors, information on several aspects of cirrhosis in Ghana is lacking. This descriptive study aimed to describe the socio-demographic and clinical characteristics of patients with cirrhosis who died at the Department of Medicine of the Korle-Bu Teaching Hospital (KBTH) in Ghana.

## Methods

### Study design and data collection

This descriptive study used secondary data from the Korle Bu Teaching Hospital Department of Medicine. The Korle-Bu Teaching Hospital is a tertiary hospital in Accra, Ghana, with a bed capacity of 1,800. It has a dedicated Gastroenterology Unit within the Department of Medicine that receives referrals from mostly the southern sector of Ghana. All files and folders of patients who died in the Department of Medicine, Korle-Bu Teaching Hospital, between the years 2014 and 2018 were retrieved from the records unit of the department. Files of patients with a confirmed diagnosis of cirrhosis and its complications were selected for the study. A file was included in the study if the diagnosis of cirrhosis was based on either of the following: 1) clinical features plus radiological findings consistent with cirrhosis prior to or during the index admission and 2) laboratory parameters (biochemical and haematological including histological confirmation from liver biopsy). Data captured from the medical records of these participants included – age, gender, duration of sickness, signs and symptoms, cause(s) of the cirrhosis (or other comorbidities), history of substance use, presence of complications, biochemical and haematological laboratory results, and pharmacological treatment received. Patients with hepatocellular carcinoma were excluded because in most of the cases, once a diagnosis of hepatocellular carcinoma was made, no efforts were made to confirm the presence and severity of underlying cirrhosis. Details of all these data outlined in the inclusion criteria were extracted and first transferred to a questionnaire and later onto Microsoft Excel 2013. Only patients with at least 80% complete data on the questionnaire were included. The reasons for exclusion included lack of age, lack of convincing data on the diagnosis of cirrhosis, lack of data on HCV/HIV or HBV and uncertainty about the outcome of the disease. Based on the small number of incomplete data excluded (not more than five patients' data), it is unlikely that the exclusion of these affected the results.

### Data analysis

Data were analysed using Microsoft Excel 2013, GraphPad Prism 7 and SPSS version 16. Fibrosis scores and indices such as AST/ALT ratio, Aspartate – platelet ratio index (APRI), FIB-4 index and Child-Pugh scores for assessing cirrhosis severity were computed.^12^–^14^ Normally distributed data were represented with mean ± standard deviation. Frequencies and percentages were used to represent categorical data.

### Ethical Considerations and consent to participate

Ethical clearance (Refs: CUC-IRB 2018/19) was obtained from the Committee of Ethical Clearance and Institutional Review Board, of the Central University (CU-IRB), Ghana.

## Results

This study obtained and analysed two hundred and forty-seven (247) patient files. The mean age of the patients was 47 years, with the age group of 30-44 years having the highest prevalence (38.9%.). The majority of the patients were males (73.3%), and the male-to-female ratio was 3:1. Table 1 shows the descriptive summary of the demographics and clinical history of the cirrhotic patients. The most common etiological factor among the patients was HBV infection (53.8%) followed by alcohol use (31.6%) and HCV infection (4.9%). The cause of cirrhosis among 21.0% of the patients was unknown or not stated. More than half (55.0%) of the patients were admitted between the second and fourth months after the start of their presenting symptoms or illness, and 67.2% died within the first two (2) weeks of admission to the unit. Majority (60.7%) of the patients were admitted once, whereas only one of them was admitted on 5 occasions. Other details can be found in Table 1

**Table 1:** Descriptive summary of sociodemographic and history of the study participants

Variable	Category	Participant n (%)
**Gender**	Male	182(73.7)
	Female	65(26.3)
**Ethnic Group**	Akan	115(46.6)
	Ewe	51(20.6)
	Ga-Dangbe	53(21.5)
	Northern*	28(11.3)
**Age (Yrs)**	15 – 29	23 (9.3)
	30 – 44	96 (38.9)
	45 – 59	72 (29.1)
	60 – 74	44 (17.8)
	> 74	12 (4.9
**Duration of Illness before** **Admission (w)**	< 1	33 (13.4)
	1 – 2	21 (8.5)
	3 – 4	27 (10.9)
	5 – 8	71 (28.7)
	12 – 16	65 (26.3)
	20 – 24	29 (11.7)
	25 – 48	1 (0.4)
**Duration of Illness** **from Admission till** **Death (w)**	< 1	120 (48.6)
	1 – 2	46 (18.6)
	3 – 4	27 (10.9)
	5 – 8	22 (8.9)
	12 – 16	8 (3.2)
	20 – 24	13 (5.3)
	25 – 48	11 (4.5)
**No. of Admissions**	Once	150 (60.7)
	Twice	78 (31.6)
	Thrice	15 (6.1)
	Fourth	3 (1.2)
	Fifth	1 (0.4)
**Prevalence of** **HBV/HCV** **Infections among Patients**	HBV	133(53.8)
	HCV	12(4.9)
**Causes of Cirrhosis**	Alcohol only	46 (18.6)
	Autoimmune	16 (6.5)
	HBV only	97(39.3)
	HCV only	2(0.8)
	Alcohol and HBV	29 (11.7)
	Alcohol and HCV	3 (1.2)
	HBV and HCV	2 (0.8)
	Unknown/Un-found	52 (21.0)

Data is presented as frequency (%, percentage). W: weeks, Yrs: Years and No is number. n: also number of.

* include: Mole-Dagbani, Mande Busanga, Gurma and Grusi. HBV; Hepatitis B virus. HCV: Hepatitis C virus.

**Figure 1:**
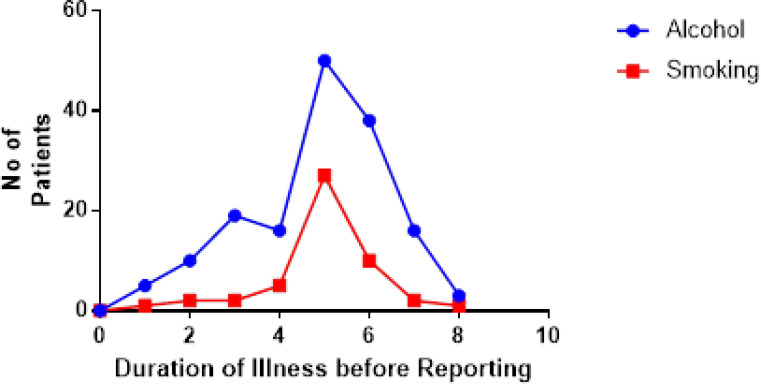
Substance use and duration of illness before patients report to health facility

**Supplementary Figure 3 (SF3):**
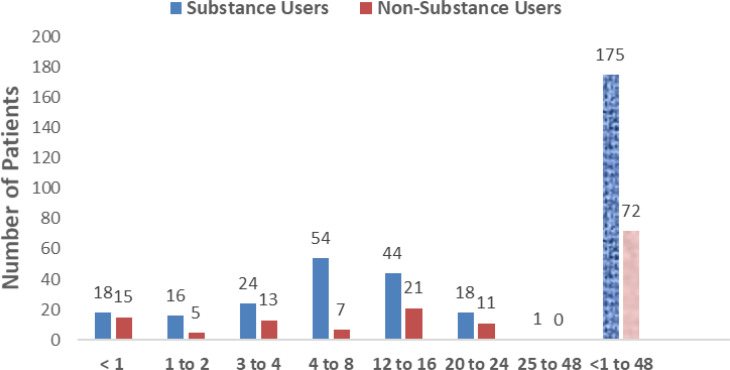
Duration of illness prior to admission between substance users and non-substance users

**Figure 2:**
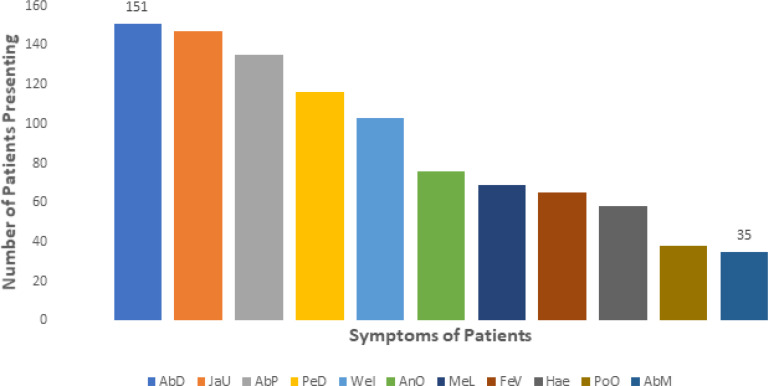
Presenting symptoms of cirrhotic patients. Abdominal distention (AbD), Abdominal Mass (AbM), Jaundice (JaU), Abdominal Pain (AbP), Pedal oedema (PeD), Weight loss (WeI), Anorexia (AnO), Melena (MeL), Fever (FeV), Haemetemesis (Hae), and Pedal Oedema (PoO).

The study examined the relationship between two life-styles – alcohol use and smoking (marijuana and/ cigarette) and the duration of illness (in weeks) prior to admission (Figure 1). More patients (63.6%) used alcohol than smoked (marijuana or cigarette) (20.2%) - for all the weeks of delay. For both lifestyles, most of the patients reported to the clinic in the 5th week of the illness (32.0% and 54.0% for alcohol and smoking, respectively). A comparison of the duration of illness prior to admission between alcohol users and smokers and non-users and non-smokers found more delays in reporting symptoms among the substance users than the latter (Supplementary Figure 3, SF3). The most common presenting symptom was abdominal distension - present in 61.1% of patients, and the least was the presence of an upper-abdominal mass in 14.2% of patients. Other presenting symptoms of the patients are shown in Figure 2).

**Supplementary Figure 1 (SF1):**
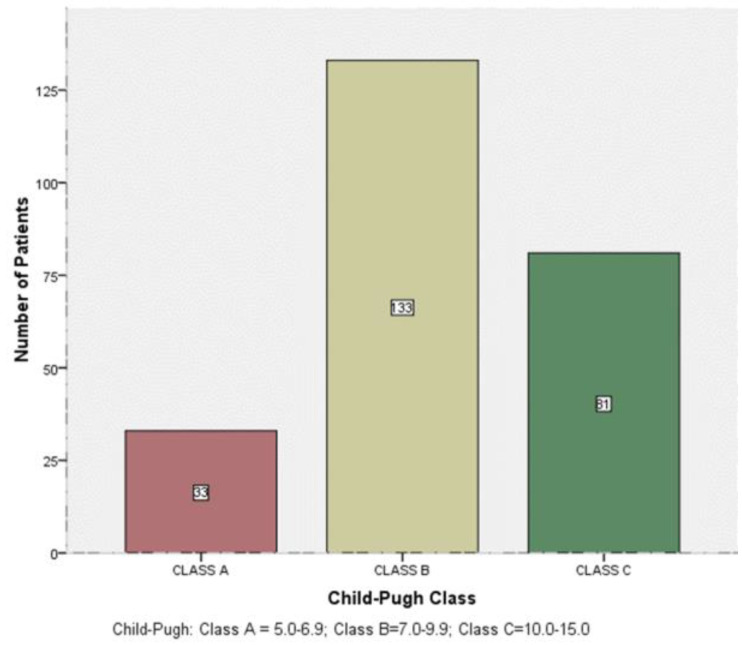
Child-Pugh Classes of the study participants

The study further analysed the patients' biochemical, haematological and fibrotic indices (Table 2). The levels of total bilirubin and the activity of AST, ALT, ALP and GGT were elevated above the baseline reference ranges. The mean total protein and platelets levels were normal, but albumin and haemoglobin levels were reduced. All the markers of fibrosis were elevated – (Table 2). The study further classified the Child-Pugh score to evaluate the severity of the cirrhosis and the likelihood of survival or death [Supplementary figure 1, SF1). More than half (53.8%) of the patients were in Class B cirrhosis, 13.4% were in class A and 32.8% were in class C (SF1).

**Table 2:** Biochemical, haematological and fibrotic indi-ces of the cirrhotic patients

Parameter (Unit)	Median (IQR)	Proportion with values outside the RR, n (%)
Total Bilirubin (µmol/l)	62.6 (28.7 -148.0)	202(82.0) α
AST (U/L)	152 (85 – 285)	225(91.0) α
ALT(U/L)	82 (49 – 117)	207(84.0) α
ALP (U/L)	252 (120 – 410)	122 (49.0) α
Total Protein (g/l)	69 (63 – 76)	42(17.0) β; 33(13.0) α
Albumin (g/l)	28 (24 -32)	150(61.0) β
GGT (U/L)	192 (82 – 367)	M:207(84.0)α,F:235(95.0)α
Hb (g/dl)	10.0 (8.0 – 11.0)	190 (77.0) β
Platelet Count (x 10^9^)	200 (131 – 267)	35(14.0) β
INR	1.7 (1.3 – 2.5)	202 (82.0) α
AST/ALT Ratio	2.0(1.1 – 3.0)	209 (85.0) α
APRI	4.2 (1.3 – 100.0)	181 (73.0) α
FIB -4 Index	4.0 (0.16 – 72.00)	209 (85.0) α
Child-Pugh	8.8 (0.0 – 5.0)	235 (95.0) α

Data is presented as Median (IQR). M: Male, F: Female, INR: International Normalized Ratio. ALT: Alanine aminotransferase, AST: Aspartate aminotransferase, ALP: Alkaline Phosphatase, GGT: Gamma-glutamyl transferase, and APRI index: Aspartate aminotransferase-to-platelet ratio index.

α Proportion above the reference range

β Proportion below the reference range.

The study also examined classes of treatments offered to the patients while on admission. Most (58.0%) of the patients were treated with antibiotics, of which 86.0% received intravenous metronidazole. In addition, 41.0%, 18.2%, 50.6% and 11.7% received analgesics, blood transfusion, lactulose, and propranolol, respectively.

The study finally analysed the common complications associated with cirrhosis in the study population (SF2). Hepatic encephalopathy was the most common (30%) followed by bleeding oesophageal varices (20%), then ascites (19%), spontaneous bacterial peritonitis, SBP, (15%), and coagulopathies (14%). The least observed complication was hepatorenal syndrome (HRS) which was observed in only 2% of the patients (SF2). Only 35(14.2%) patients reported 1 complication, the rest had 2 or more complications.

## Discussion

This study examined the sociodemographic and medical characteristics of cirrhotic patients who were admitted and died at the Gastroenterology Unit of the Department of Medicine, KBTH, Accra, Ghana. Most cirrhotic patients were males between thirty to fifty years old. Duah *et al.* reported similar findings, and a global survey also reported a higher male: female ratio and higher prevalence in middle age among patients with cirrhosis.^2^,^4^,^5^,^6^,^15^

In addition to the known higher prevalence of liver disease in males, longer delays in seeking medical care in males than in females may account for the higher male mortality. The finding that two-thirds of the patients are in their middle and reproductive ages has been previously reported by other studies.^16^,^17^

This is the first study reporting the distribution of cirrhosis among ethnic groups in Ghana. The Akan majority finding from our study reflects the national, ethnic distribution. It is consistent with the findings of Archampong & Nkrumah (2016), who reported similar distributions among HBV-infected patients.^18^ Compared to the national distribution, the Ga-Dangbes are over-represented in our study consisting of more than twice the national percentage distribution. This is possibly because the study was done in a predominantly Ga-Dangbe community and in Accra - where four (4) of every five (5) Ga-Dangbes reside.^19^ The reverse (of the Ga-Dangbe reason) might explain the lower representation of ‘Northern’ ethnic groups among the study participants compared with the national structure.^20^

The most prevalent causes of cirrhosis in this study were HBV infections and alcohol use, a finding consistent with a worldwide study on the global burden of cirrhosis.^2^ Similar findings have been reported in other countries where HBV infection is endemic, like Ghana.^2^,^4^,^6^,^9^,^21^ Although HBV is endemic in Ghana, at birth dose vaccination of newborns remains to be implemented, organised screening for adults except pregnant women is lacking, and evaluation and treatment of chronically infected persons can be expensive and not fully covered by the National Health Insurance Scheme. These factors probably account for the high prevalence of HBV infection among cirrhotic patients. Long-term HBV infection and alcohol misuse are characterised by repeated inflammation and repair of the liver hepatocytes, eventually leading to fibrosis and cirrhosis. Autoimmune liver cirrhosis is caused by autoimmune liver diseases (AILD). AILDs are caused directly by autoimmune hepatitis (AIH) types 1 and 2 and indirectly by primary sclerosing cholangitis and primary biliary cirrhosis. However, among one-fifth of the participants, cirrhosis's cause(s) was not found. In these patients, no evidence for consideration for NAFLD nor results for tests for uncommon causes for cirrhosis like Wilson's disease, alpha 1 antitrypsin, haemochromatosis and other genetic tests^22^ were found. This highlights a challenge in the workup of patients with cirrhosis and is likely due to resource constraints.

These patients were admitted because of their presenting complaints, which made them sick. These symptoms were confirmed as one form of decompensation or the other subsequently. Most patients reported symptoms of 8-24 weeks duration, and the majority also died after a relatively short (i.e., two weeks) hospital stay. The delays in seeking healthcare may be because of delays in referrals as KBTH is a referral centre, and all patients must be referred to assess care there. However, other reasons for delays in seeking healthcare, such as financial constraints, lack of medical insurance coverage, poor under-standing of the disease, beliefs in traditional medicine as a cure for many diseases and lack of support from family and friends, are prevalent in Ghana and could have been contributory.^23^ With such delays, it is likely that cases managed at KBTH either had severe or advanced disease hence the rapid progression to death.

The study also confirms the severity of liver disease disease in the study population; most of the patients were in Child Pugh Class B. Duah *et al* found most patients in Class C.^6^ The presenting symptoms were not different from those reported by similar studies in Ghana.^4^–^6^ One of the causes of abdominal distension, which was the most common presenting symptom, was ascites, and this is associated with increased mortality among cirrhotic patients.^25^

This study's reported prevalence of complications are similar to reports from other centres in Ghana and elsewhere.^4^–^6^,^26^,^27^ The presence of these complications are often markers of the decompensated state of cirrhosis and often the reason for admission that ultimately led to death in our patients. Compensated cirrhosis is largely asymptomatic. The pattern of liver test abnormalities are similar to that reported by Duah *et al.*, (2021) where some abnormal laboratory parameters were associated with mortality.^6^,^28^ The mean albumin levels were expectedly low as it measures the synthetic function of the liver, which is reduced in cirrhosis and has been similarly reported in other studies.^5^,^29^ The low haemoglobin (Hb) found in our study is consistent with high prevalence of variceal bleed in this cohort of patients. Other reasons for this may include loss of synthetic function of the liver, poor nutrition among patients with cirrhosis, and bone marrow suppression.^30^,^31^ Surprisingly, thrombocytopaenia (low platelets count) was not observed from our study. This contrasts with several others reports on platelet count in cirrhosis. 32,33 The reasons for this disparity are unclear and may warrant further studies.

The AST/ALT ratio, APRI, Child-Pugh and FIB -4 Index scores were elevated in 85.0%, 73.0%, 95.0% and 85.0% of the patients, respectively, like what has been previously reported by other studies.^34^,^35^ The high prevalence of abnormal scores is a possible confirmation of their usefulness in identifying cirrhosis within a population. Further studies are needed to validate these scores in the Ghanaian population.

Our study also showed that most of the patients dying from cirrhosis were in Child-Pugh Class B. This indicates less severe liver disease than was reported by Duah et al (Class C), who reported on all cirrhotic patients on admission.

This finding suggests that the reasons for death may be beyond just the severity of the liver disease. Identifying these factors in further studies and addressing them at admission may help to avert mortalities in future.

Pharmacological treatment in decompensated cirrhosis targets the identified complications and the underlying cause. Metronidazole and lactulose are part of the standard of care for patients with hepatic encephalopathy 36,37, propranolol for variceal bleed 38, iv antibiotics for SBP and diuretics for ascites. The findings from this study confirm lactulose and oral metronidazole as the preferred choice for clinicians treating encephalopathy. Diuretic use was not found in the medication list of our study population, especially when ascites was present in 19.0% of them. The reasons for the non-use of diuretics require further studies. Also, since chronic HBV infection was the most prevalent aetiological factor, we expected to find a definite treatment for this, but that was missing. This is surprising because cirrhosis is an indication for anti-HBV therapy and the Hepatitis Guidelines for the care of patients recommends treatment for this group. Furthermore, at least tenofovir is readily available for the treatment of this group, although patients must pay about 20 US dollars equivalent out of pocket for it. It is possible that among the studied patients, their presentation was late and the prognosis at admission was poor; hence definite treatment was not considered prudent at the time.

The retrospective nature of this study was a limitation to the quality of the data collected. Not all data was available for all the patients, however, percentages were calculated using only the total number with the most variables. This was also a single-centre study; hence the data may not represent the national situation. Additionally, endoscopy and variceal band ligation services are available at KBTH and patients with variceal bleeding likely received this intervention, but this was not captured as treatment.

## Conclusion

Deaths from cirrhosis at the Korle Bu Teaching Hospital are mostly of young males infected with chronic hepatitis B. Since chronic hepatitis B is largely preventable through measures such as immunisation and treatment of chronic infections, these cirrhosis deaths can be prevented. Efforts to mitigate the burden of cirrhosis on the Ghanaian population should include efforts by the government to provide resources to implement at birth dose HBV vaccination and policy direction to allow the inclusion of HBV evaluation and drugs on the National Health Insurance benefits package. Additionally, educating the cirrhosis patient group to avoid delays in seeking healthcare for their symptoms will prevent some of the mortalities.

## Figures and Tables

**Supplementary Figure 2 (SF2):**
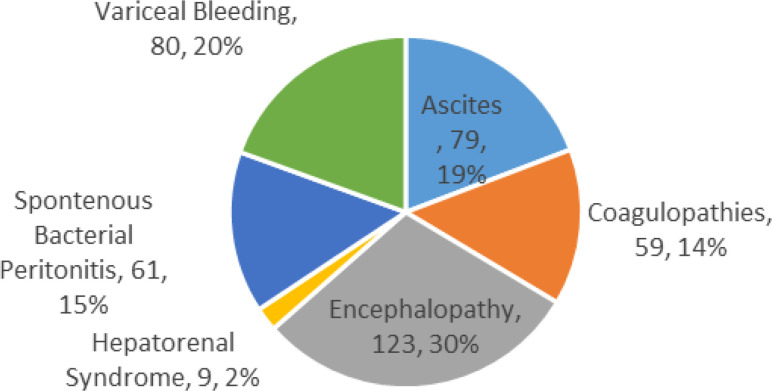
Complications among cirrhosis patients
